# Multicenter epidemiology of *Stenotrophomonas maltophilia* bloodstream infections in Indian ICUs: building digital surveillance network

**DOI:** 10.3389/fmicb.2025.1725629

**Published:** 2025-12-04

**Authors:** Parul Singh, M. Nizam Ahmed, Ashish Kumar Srivastava, Arpan Kumar Thakur, Rasna Parveen, Mamta Puraswani, Subodh Kumar, Sushma Sagar, Kapil Dev Soni, Richa Aggarwal, Ashish Bindra, Keshav Goyal, Kamran Farooque, Arunaloke Chakrabarti, Camilla Rodrigues, Veeraraghavan Balaji, Pallab Ray, Manisha Biswal, Neelam Taneja, Archana Angrup, Chand Wattal, Vimala Venkatesh, Nandini Sethuraman, Sanjay Bhattacharya, Vibhor Tak, Bijayini Behera, Vinaykumar Hallur, Raja Ray, Shivaprakash M. Rudramurthy, Inderpaul Sehgal, Sanjeev K. Singh, Sharmila Sen Gupta, Chiranjay Mukhopadhyay, Joy Sarojini Michael, Bashir Ahmad Fomda, Tadepalli Karuna, Vijayshri Deotale, Amber Prasad, Kanne Padmaja, Vijeta Bajpai, Reema Nath, Renu Gur, Sheela Devi, Shalini Malhotra, Rajni Gaind, Ranjana Devi Khuraijam, Rajni Sharma, Summaiya Mullan, John Antony Jude Prakash, Hema Paul, Priscilla Rupali, Sheetal Verma, Sangita Rajdev, Neeraj Goel, Juliah Chelliah, Satyam Mukherjee, Aparna Sonowal, Veena Kumari, Prachi Verma, Vandana KE, Manisha Subrao Mane, Tapan Majumder, Kamini Walia, Purva Mathur

**Affiliations:** 1 Department of Laboratory Medicine, JPNA Trauma Center, All India Institute of Medical Sciences, Delhi, India; 2 Department of Surgery, JPNA Trauma Center, All India Institute of Medical Sciences, Delhi, India; 3 Department of Critical and Intensive Care, JPNA Trauma Center, All India Institute of Medical Sciences, Delhi, India; 4 Department of Neuroanaesthesia, JPNA Trauma Center, All India Institute of Medical Sciences, Delhi, India; 5 Department of Neuroanaesthesia and Critical Care, JPNA Trauma Center, All India Institute of Medical Sciences, Delhi, India; 6 Department of Microbiology, Postgraduate Institute of Medical Education and Research, Chandigarh, India; 7 Department of Microbiology, P.D. Hinduja National Hospital, Mumbai, India; 8 Department of Clinical Microbiology, Christian Medical College and Hospital, Vellore, India; 9 Institute of Clinical Microbiology and Immunology, Sir Ganga Ram Hospital, Delhi, India; 10 Department of Microbiology, King George's Medical University, Lucknow, India; 11 Department of Microbiology, Apollo Hospital, Chennai, India; 12 Department of Microbiology, Tata Medical Center, Kolkata, India; 13 Department of Microbiology, All India Institute of Medical Sciences, Jodhpur, India; 14 Department of Microbiology, All India Institute of Medical Sciences, Bhubaneswar, India; 15 Department of Microbiology, Institute of Post Graduate Medical Education and Research, Kolkata, India; 16 Department of Microbiology, Amrita Institute of Medical Sciences, Kochi, India; 17 Department of Microbiology, Kasturba Medical College, Manipal, India; 18 Department of Microbiology, Sher-i-Kashmir Institute of Medical Sciences, Srinagar, India; 19 Department of Microbiology, All India Institute of Medical Sciences, Bhopal, India; 20 Department of Microbiology, Mahatma Gandhi Institute of Medical Sciences, Sevagram, India; 21 Department of Microbiology, All India Institute of Medical Sciences, Rishikesh, India; 22 Department of Microbiology, Nizam's Institute of Medical Sciences, Hyderabad, India; 23 Department of Microbiology, Homi Bhabha Cancer Hospital and Mahamana Pandit Madan Mohan Malaviya Cancer Centre, Varanasi, India; 24 Department of Microbiology, Assam Medical College and Hospital, Dibrugarh, India; 25 Department of Microbiology, Dr. Baba Saheb Ambedkar Hospital, Delhi, India; 26 Department of Microbiology, Pondicherry Institute of Medical Sciences, Pondicherry, India; 27 Department of Microbiology, Dr. Ram Manohar Lohia Hospital and PGIMER, Delhi, India; 28 Department of Microbiology, Safdarjung Hospital, Delhi, India; 29 Department of Microbiology, Regional Institute of Medical Sciences, Imphal, India; 30 Department of Microbiology, Sawai Man Singh Medical College, Jaipur, India; 31 Department of Microbiology, Government Medical College, Surat, India; 32 Department of Neuro-Microbiology, National Institute of Mental Health and Neuro-Sciences, Bengaluru, India; 33 Department of Microbiology, Mahatma Gandhi Medical College and Hospital, Jaipur, India; 34 Department of Microbiology, ESIC Medical College and Hospital, Hyderabad, India; 35 Department of Microbiology, Government Medical College, Agartala, India; 36 Division of Epidemiology and Communicable Diseases, Indian Council of Medical Research, Delhi, India

**Keywords:** healthcare-associated infections, *Stenotrophomonas maltophilia*, BSIs, bloodstream infections, surveillance

## Abstract

**Background:**

To investigate the geospatial epidemiology, clinical features, treatment patterns, and antimicrobial resistance (AMR) trends of *Stenotrophomonas maltophilia* bloodstream infections (BSIs) in Indian intensive care units (ICUs) participating in a standardized healthcare-associated infection (HAI) surveillance program from 2017 to 2024.

**Methods:**

This retrospective, multicentric study analyzed surveillance data from 54 ICUs across India. Standardized HAI definitions and protocols were applied to characterize infection types, clinical outcomes, and antimicrobial susceptibility.

**Results:**

A total of 271 *S. maltophilia* isolates were identified, with the highest burden in 2023–24 (*n* = 76, 28.0%). Central line-associated BSIs (CLABSIs) predominated (64.9%), though their proportion decreased over time, with non-CLABSIs rising from 7.4% (2017–18) to 42.1% (2023–24). Mortality was highest in secondary BSIs (60%), followed by CLABSIs (50.3%) and non-CLABSIs (36.4%). The median ICU stay for CLABSI patients was 21 days. No significant associations were observed between infection type and time to infection or length of stay. High resistance was observed to tobramycin (92%), amikacin (80%), and piperacillin-tazobactam (70%), while trimethoprim-sulfamethoxazole (64.7–94.7%), levofloxacin (93%), and minocycline (94.1%) retained activity.

**Conclusion:**

*S. maltophilia* represents a significant ICU pathogen in India, underscoring the urgent need for genomic surveillance and resistance-guided therapeutic strategies.

## 1 Introduction

*Stenotrophomonas maltophilia* is an emerging opportunistic pathogen increasingly recognized in healthcare settings, particularly among critically ill and immunocompromised patients. This non-fermenting, Gram-negative bacillus is ubiquitous in the environment and is notorious for its intrinsic resistance to multiple antibiotics, including carbapenems, and its association with healthcare-associated infections such as bloodstream infections (BSIs) (Baidya et al., 2019; Adegoke et al., 2017; Sezen et al., 2025). Its ability to form biofilms and its multidrug-resistant (MDR) nature pose significant treatment challenges, contributing to high morbidity and mortality rates (Nayyar et al., 2017; Chen et al., 2025). BSIs caused by *S. maltophilia* are particularly prevalent in intensive care units (ICUs), where indwelling devices like central venous catheters are major risk factors for central line-associated bloodstream infections (CLABSIs) (Huang et al., 2024; Mukhopadhyay et al., 2003).

The global epidemiology of *S. maltophilia* BSIs shows a rising incidence, particularly in ICU, trauma, and oncology settings, with the COVID-19 pandemic exacerbating infection rates due to prolonged hospitalisation and invasive procedures (Quang et al., 2025; Gupta et al., 2018; Song et al., 2023). In India, *S. maltophilia* is an emerging concern in tertiary care centres, driven by high patient acuity and widespread use of invasive devices (Brooke, 2021; Varshini et al., 2022). Antimicrobial resistance further complicates management, with increasing resistance to traditional agents like trimethoprim-sulfamethoxazole (TMP SMX) and variable susceptibility to alternatives such as levofloxacin and tigecycline (Mojica et al., 2022; Banar et al., 2023). This study investigates the Geospatial epidemiology, clinical characteristics, treatment patterns, and AMR trends of *S. maltophilia* BSIs across multiple healthcare centers of India from 2017 to 2024, analyzing 271 isolates to elucidate infection sources, patient outcomes, and resistance profiles in critical care settings.

## 2 Methodology

This retrospective, multicentre, hospital-based surveillance study analyzed cases of *S. maltophilia* infections identified in ICUs over a period of seven years. The focus was on bloodstream infections (BSIs) among ICU patients during this timeframe.

The study included data from 54 tertiary-care hospitals across India, each with dedicated infection prevention and control teams and accredited microbiology laboratories participating in the HAI Surveillance Network of India (www.haisindia.com or https://api.haisindia.com). Data from cases of BSI caused by *S. maltophilia* were collected between May 2017 to April 2024. Details of the participating centres that reported *S. maltophilia* in BSIs are provided in Supplementary Table S1. Patient follow-up extended through the hospital stay and concluded at discharge/death/transfer-out, whichever occurred first.

### 2.1 Participants

Each hospital taking part in the study included a minimum of one ICU for adult medical patients, one for adult surgical patients, and one paediatric ICU in their bloodstream infection monitoring. To maintain consistency, hospitals aligned each ICU they enrolled with standard ICU categories defined by the network’s coordinating team.

Following enrolment, hospital surveillance teams completed a two-day induction workshop organized by the network coordinators. To reinforce quality and consistency, follow-up training sessions were conducted twice yearly during investigator meetings and during on-site visits by the coordinating staff.

Dedicated teams at each site carried out active tracking of bloodstream infections within their designated ICUs. For every identified BSI, staff filled out a standardized case report form that collected patient demographics, clinical details, isolated pathogens with their routine antimicrobial susceptibility profiles, and the patient’s final outcome.

### 2.2 Procedures

A dedicated web-based platform was designed for reporting, compiling, and analyzing surveillance data (www.haisindia.com or https://api.haisindia.com). Participating hospitals submitted records of bloodstream infections that met the study’s case definitions, along with denominator data, through this online system at least once a month. Personal identifiers were replaced with unique case number codes to maintain confidentiality. Using the information provided in each case report form, the platform automatically sorted BSI cases into relevant categories: central line-associated bloodstream infection (CLABSI), primary BSI unrelated to a central line, or secondary BSI. While each hospital could access and analyze its own data through the system, only the network coordination team had access to view the combined dataset, with all identifiers removed.

For each BSI case, *S. maltophilia* isolates and corresponding antimicrobial susceptibility test (AST) results were reported using data provided by hospital microbiology laboratories. Laboratories employed their standard bacterial isolation and identification techniques. Sensitivity testing methods included automated systems (Vitek-2) or conventional manual techniques (disk-diffusion method). The antimicrobial sensitivity testing was performed following Clinical and Laboratory Standards Institute (CLSI) guidelines (updated CLSI version) and breakpoints. Reported pathogens were compiled across all ICUs and ranked by frequency, with AST results summarized for the organism for the entire network.

The network coordination team routinely reviewed submissions from each hospital every month to detect and correct any reporting gaps. To ensure quality and consistency in surveillance practices across all sites, periodically updated standard operating procedures (SOPs) were shared with the network hospitals. The coordination team (at JPNATC, AIIMS, New Delhi) reviewed all the submitted data on the portal to identify and address any discrepancies. The coordination team conducted at least one site visit to each hospital, preferably soon after the start of data collection. During these visits, standardized checklists were used to assess adherence to network protocols, identify areas for improvement, and provide targeted feedback to local surveillance teams. Regular training sessions, including workshops and site visits, were conducted to ensure the quality of data collection. In addition to regular training, refresher training was given during the network investigators’ meetings, held twice a year.

**Figure 1:**
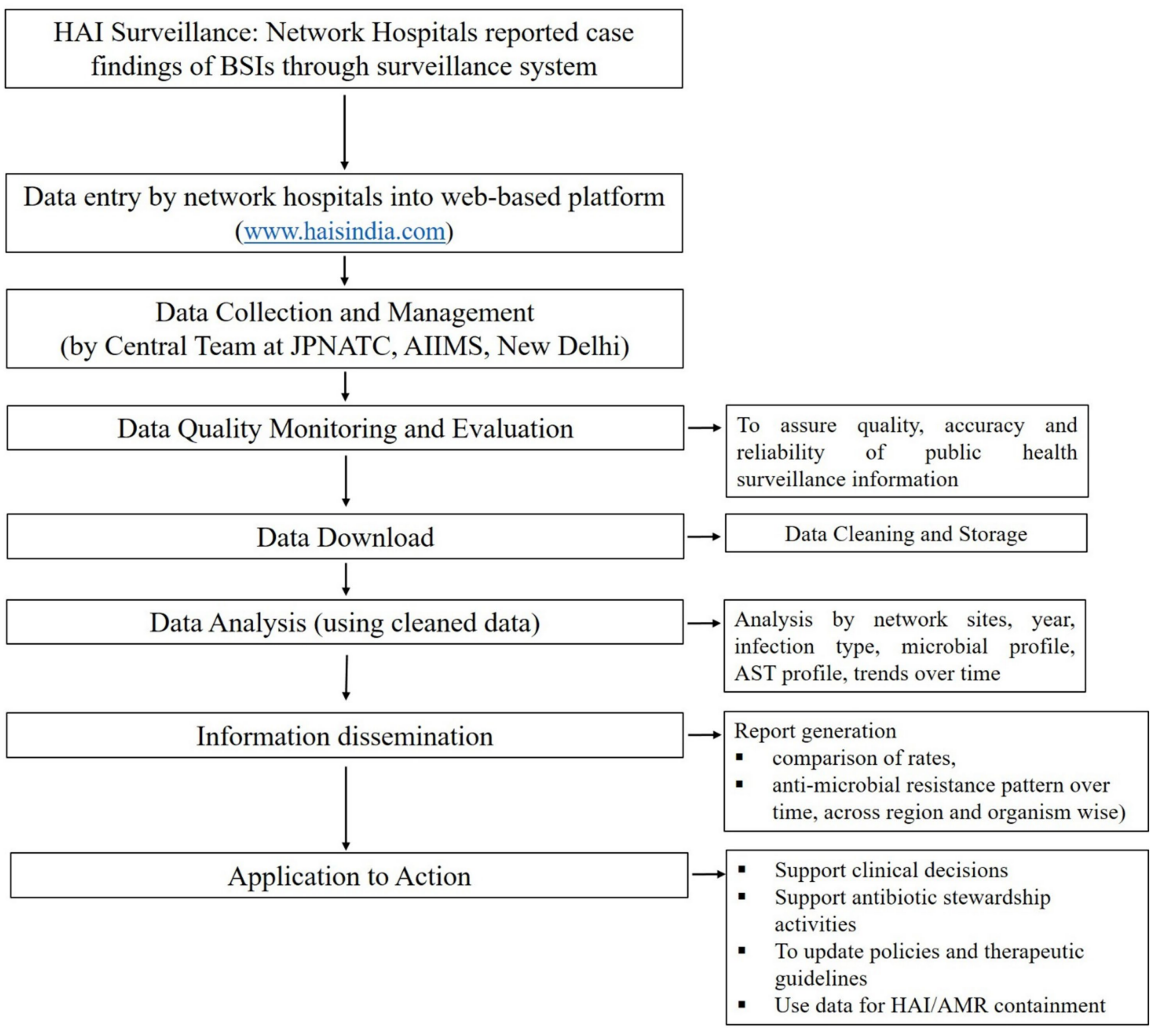
Data flow and processes of HAI surveillance through HAI surveillance database.

Data flow and processes of HAI Surveillance through HAI Surveillance database shown in Figure 1.

### 2.3 Inclusion and exclusion criteria

#### 2.3.1 Inclusion criteria

The patient must be hospitalised in the ICU (surveillance unit) for more than 2 calendar days. The BSI should occur more than 2 calendar days after admission to ICU (surveillance unit). No BSI should have been reported in the past 14 days.

#### 2.3.2 Exclusion criteria

Patients who were not admitted in the ICUs, BSI occurring in less than 2 calendar days from the date of admission to the surveillance unit (ICU).

### 2.4 Definitions

#### 2.4.1 CLABSI primary BSI

Primary BSI in which a central line was in place for more than 2 calendar days on the date of infection or the central-line has been removed on the day or one day prior to the date of infection (Mathur et al., 2022).

#### 2.4.2 Non-CLABSI primary BSI

Primary BSI that occur without a central line in place or after the central line has been removed two days before infection and not associated with infection at another body site are called non-central line primary bloodstream infections (non-CLABSIs) (Quang et al., 2025; Srivastava et al., 2025).

#### 2.4.3 Secondary BSI

BSI in which all organisms identified in blood culture are also identified from other body sources either 7 days prior or 14 days after the date of infection (Mathur et al., 2022).

### 2.5 Statistical analysis

Statistical analysis was done using R software package version 4.4.0 and parameters such as patient characteristics, case events and isolates were investigated. Descriptive data are presented, as *n* (%) where n is either the number of patients or organisms. For age, length of stay (LOS) and time to infection (TTI: duration between ICU admission and date of event), median and IQR was calculated. Fisher’s exact test was done to analyse the association between categorical variables (TTI and LOS) and infection types (outcome).

## 3 Results

**Figure 2:**
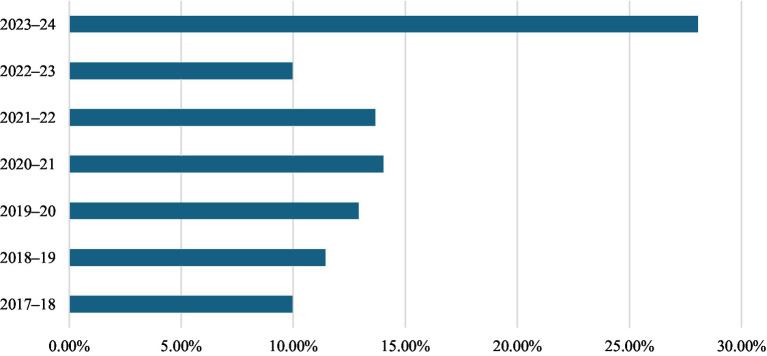
Year-wise isolation from bloodstream (2017–2024).

This retrospective, multicentre observational study was conducted across 54 healthcare centres. A total of 271 *S. maltophilia* were identified from BSI events between 2017 and 2024. The annual number of isolates showed an increasing trend, with the highest count observed in 2023–24 (*n* = 76, 28.04%), compared to 27–38 (9.96–14.02%) per year in previous years as shown in Figure 2.

Central line-associated bloodstream infections (CLABSIs) were the predominant source, accounting for 64.9% (*n* = 176/271) of isolates, followed by non-CLABSIs (27.3%, *n* = 74) and secondary BSIs (7.7%, *n* = 21). In our HAI surveillance, secondary BSIs were classified as per the primary source of infection. The primary source included respiratory infections such as VAP, urinary tract infections (such as CAUTI and non-CAUTI), surgical site infections or skin and tissue infections. Classification of ICUs reporting *S. maltophilia* infections in the HAI Surveillance network detailed trends are presented in the Supplementary Table S2.

### 3.1 Trend of *S. maltophilia* BSIs

Over the study period, the proportion of CLABSI-associated isolates declined from 81.5% in 2017–18 to 55.3% in 2023–24, while non-CLABSI isolates increased from 7.4 to 42.1%. Isolation of *S. maltophilia* from secondary BSIs remained consistently low, ranging from 2.6 to 13.5% annually.

Region-wise distributions of BSI events caused by *S. maltophilia* are depicted in Supplementary Table S3 and year-wise distribution of BSI cases is shown in Supplementary Table S4.

During the COVID-19 pandemic period, notable shifts in BSIs case were observed; the detailed trends are presented in the Supplementary Table S4.

Of the 235 patients included, 42% (*n* = 98) were female and 58% male (*n* = 137), with a median age of 42 years (range: 22–59). Among subgroups, CLABSI patients had a slightly higher median age (43 years), while secondary BSI patients were 53 years. The 14 day all-cause mortality ranged from 29% (*n* = 19, non-CLABSI) to 37% (*n* = 55, CLABSI), while final outcome mortality (outcome at the end of hospitalization) was highest in secondary BSI cases 75% (*n* = 12), compared to CLABSI 53% (*n* = 75) and non-CLABSI 39% (*n* = 24). However, the mortality was not attributable to BSIs event since other ICU/patients related factors also contributed to mortality. The median duration of stay in the unit was longest for CLABSI patients (21 days), and time from admission to BSI diagnosis ranged from 6 to 9 days across groups (Table 1).

**Table 1:** Demographic data of BSIs caused due to *Stenotrophomonas maltophilia.*

Characteristics	Overall (*N* = 235)	CLABSI (*n* = 149)	Non-CLABSI (*n* = 66)	Secondary BSI (*n* = 20)
Gender				
Female	98 (41.7%)	62 (41.6%)	27 (41.5%)	9 (45%)
Male	137 (58.3%)	87 (58.4%)	39 (60%)	11 (55%)
Age {median (Q1, Q3)}	42 (22, 59)	43 (24, 60)	35 (18, 58)	53 (26, 58)
14-day outcome				
Died	80 (34%)	55 (36.9%)	19 (28.8%)	6 (30.0%)
Discharged	29 (12.3%)	13 (8.7%)	15 (22.7%)	1 (5.0%)
LAMA (Left against medical advice)	7 (3.0%)	5 (3.4%)	1 (1.5%)	1 (5.0%)
Still in surveillance unit	78 (33.2%)	47 (31.5%)	21 (31.8%)	10 (50.0%)
Transferred to other hospital	3 (1.3%)	2 (1.3%)	1 (1.5%)	0
Transferred to other unit/ward within the hospital	37 (15.7%)	26 (17.4%)	9 (13.6%)	2 (10.0%)
Unknown	1 (0.4%)	1 (0.7%)	0	0
Final outcome				
Died	111 (47.2)	75 (50.3%)	24 (36.4%)	12 (60.0%)
Discharged	91 (38.7)	54 (36.2%)	34 (51.5%)	3 (15.0%)
LAMA (Left against medical advice)	12 (5.1)	9 (6.0%)	2 (3.0%)	1 (5.0%)
Transferred to other hospital	4 (1.7)	3 (2.0%)	1 (1.5%)	0
Unknown	17 (7.2)	8 (5.4%)	5 (7.6%)	4 (20.0%)
Duration of stay in unit {median (Q1, Q3)}	19 (13, 33)	21 (14, 37)	14 (11, 26)	15 (12, 23)
Duration between date of admission and date of event {median (Q1, Q3)}	9 (5, 15)	9 (6, 16)	6 (4, 13)	8 (5, 18)

CLABSI, central-line associated primary bloodstream infection; Non-CLABSI, non-central-line associated primary bloodstream infection.

### 3.2 Distribution of TTI and LOS categories

Time to infection (TTI) and length of stay (LOS) were categorized to explore their distribution across CLABSI, non-CLABSI, and secondary BSI events. While variations were observed across groups, no statistically significant associations were identified (*p* = 0.469 for TTI; *p* = 0.079 for LOS). Distributions are provided in the table below (Table 2).

**Table 2:** Distribution of TTI and LOS categories across various BSI classifications (Event-Level).

Category	Overall (*n* = 235)	CLABSI (*n* = 149)	Non-CLABSI (*n* = 66)	Secondary BSI (*n* = 20)	*p*-value*
TTI category					0.469
<=7 days	100	56 (56.0)	35 (35.0)	9 (9.0)	
8–14 days	74	51 (68.9)	18 (24.3)	5 (6.8)	
15–21 days	22	14 (63.6)	6 (27.3)	2 (9.1)	
>21 days	39	28 (71.8)	7 (17.9)	4 (10.3)	
LOS category					0.079
<=7 days	23	10 (43.5)	11 (47.8)	2 (8.7)	
8–14 days	76	43 (56.6)	23 (30.3)	10 (13.2)	
15–21 days	35	25 (71.4)	7 (20.0)	3 (8.6)	
>21 days	101	71 (70.3)	25 (24.8)	5 (5.0)	

*Fisher’s Exact Test; TTI, time to infection; LOS, length of stay; CLABSI, central-line associated primary bloodstream infection; Non-CLABSI, non-central-line associated primary bloodstream infection.

### 3.3 Antimicrobial resistance patterns

Among the 271 isolates of *Stenotrophomonas maltophilia* from bloodstream infections, antimicrobial susceptibility testing showed variable resistance patterns across different drug classes.

Fluoroquinolones generally remained effective, particularly levofloxacin (93% susceptible) colistin (71.4% susceptible) and tigecycline (88% susceptible) demonstrated relatively preserved activity (Table 3). Region-wise trend of resistance pattern to various drugs is depicted in Supplementary Table S5.

**Table 3:** AST trend of *Stenotrophomonas maltophilia* against a wide variety of drugs over a period of 7 years (2017–2024).

Year	Total isolates	Trimethoprim/Sulfamethoxazole		Minocycline		Levofloxacin		Tigecycline		Colistin	
		TESTED	S	TESTED	S	TESTED	S	TESTED	S	TESTED	S
			(%)		(%)		(%)		(%)		(%)
2017–18	27	19	18 (94.7)	8	8 (100)	23	23 (100)	6	5 (83.3)	1	1 (100)
2018–19	31	17	11 (64.7)	14	12 (85.7)	29	26 (89.7)	3	3 (100)	0	0
2019–20	35	27	24 (88.9)	13	13 (100)	31	28 (90.3)	0	0	8	5 (62.5)
2020–21	38	29	24 (82.8)	20	19 (95.0)	34	31 (91.2)	2	2 (100)	3	2 (66.7)
2021–22	37	29	25 (86.2)	20	20 (100)	34	33 (97.1)	5	4 (80.0)	2	2 (100)
2022–23	27	12	10 (83.3)	20	20 (100)	25	21 (84.0)	1	1 (100)	0	0
2023–24	76	50	41 (82.0)	68	64 (94.1)	68	64 (94.1)	0	0	0	0
Total	271	183	153	163	156	244	226	17	15	14	10

## 4 Discussion

The study identified 271 *S. maltophilia* isolates from BSIs across multiple ICU settings (*n* = 86) from 2017 to 2024, with an increase in isolates over time, peaking at 76 (28.0%) in 2023–24. This rising trend, coupled with diverse infection sources and high mortality rates, underscores the growing clinical significance. *S. maltophilia* has emerged as a significant healthcare-associated pathogen, particularly in ICU settings, with increasing incidence reported across Europe, Asia, and Latin America (Koh et al., 2025; Erinmez et al., 2024; Pfaller et al., 2023). A previous study from Europe reported an incidence of 0.5–1.5 per 10,000 patient-days in ICUs, driven by prolonged hospitalizations, mechanical ventilation, and central venous catheters (Tanuma et al., 2025). In Asia, particularly Singapore and Japan, *S. maltophilia* BSIs are prevalent among immunocompromised and trauma patients, with central venous catheters identified as a primary risk factor (Bostanghadiri et al., 2024; Rajkumari et al., 2015). It is also associated with hematologic malignancies and post-surgical complications (Guerci et al., 2019). The COVID-19 pandemic increased *S. maltophilia* infections, with a previous study from Turkey noting a surge in 2020–2021, attributed to prolonged ICU stays and corticosteroid use (Sapula et al., 2024). This aligns with the current study’s finding of 11 isolates (4.1%) in COVID-specific ICUs, primarily in 2020–21, with no isolates in 2023–24, possibly reflecting reduced COVID-related hospitalizations or improved infection control (Gupta et al., 2018; Sapula et al., 2024). The global rise in *S. maltophilia* infection emphasizes the need for robust surveillance and infection prevention strategies (Erinmez et al., 2024; Patil et al., 2018).

In India, *S. maltophilia* BSIs are increasingly reported in tertiary care centres, particularly in medical, surgical, and trauma ICUs (Mosiun et al., 2025; Li et al., 2023). A previous study from North India documented *S. maltophilia* as a significant cause of Gram-negative BSIs, with a prevalence of 5–10% in ICUs (Gautam et al., 2015). The current study’s findings of 271 isolates, with medical (22.9%) and medical/surgical (21.8%) ICUs as primary sources, align with these national trends (Srivastava et al., 2022). Trauma units (11.4%) and neonatal ICUs (4.4%) also contributed significantly, consistent with a previous study highlighting the organism’s prevalence in these high-risk populations due to invasive procedures and prolonged hospitalizations (Sethi et al., 2020). The increase in isolates from 27–38 annually (2017–2022) to 76 in 2023–24 mirrors national reports of rising *S. maltophilia* infections, potentially driven by improved diagnostics or higher patient acuity (Patterson et al., 2020). The decline in CLABSI-associated isolates (from 81.5% in 2017–18 to 55.3% in 2023–24) and the rise in non-CLABSI isolates (from 7.4 to 42.1%) suggest evolving infection patterns, possibly due to enhanced catheter care or increased recognition of alternative sources like respiratory or intra-abdominal infections, as noted in a previous study from South India (Hase et al., 2024).

The patient cohort (*n* = 234) was predominantly male (59%), with a median age of 42 years, consistent with a previous study reporting a male predominance (Batra et al., 2017). Secondary BSI patients had a higher median age (53 years) compared to CLABSI (43 years) and non-CLABSI (35 years) patients, likely reflecting comorbidities or immunosuppression, as older patients are more prone to secondary infections from sources like pneumonia (Guerci et al., 2019; Sader et al., 2025). CLABSIs dominated (65% isolates), corroborating the role of central venous catheters as a primary risk factor (Huang et al., 2024; Bostanghadiri et al., 2024; Parveen et al., 2025). The 14-day all-cause mortality rate was highest for CLABSI patients (37%), while a final fatal outcome (outcome at the end of hospitalization) was highest for secondary BSIs (75%), aligning with a previous study reporting high mortality in secondary infections due to underlying conditions (Fratoni et al., 2021). The median duration of stay was longest for CLABSI patients (21 days), highlighting the challenges of addressing catheter-associated infections (Huang et al., 2024; Bostanghadiri et al., 2024). The duration from admission to BSI diagnosis (6–9 days) indicates that they were all acquired in ICUs, aligning with the organism’s hospital adaptations (Mosiun et al., 2025; Tamma et al., 2022). The lack of significant associations between time to infection (TTI) and length of stay (LOS) categories (*p* = 0.469 and *p* = 0.079, respectively) indicates that multiple factors, such as comorbidities and ICU practices, influence these parameters (Hase et al., 2024; Veeraraghavan and Walia, 2025).

Trimethoprim-sulfamethoxazole (TMP-SMX) remains the first-line agent, with our study reporting susceptibility of 64.7–94.7% (2017–2024), though a decline to 82.0% in 2023–24 aligns with a previous study noting reduced efficacy (Nayyar et al., 2017; Mukhopadhyay et al., 2003; Veeraraghavan and Walia, 2025). Levofloxacin (93% susceptible) and minocycline (94.1% susceptible at ≤4 mg/L) are key alternatives, supported by a previous study highlighting levofloxacin’s favourable pharmacodynamics (Quang et al., 2025). However, the 2024 CLSI revision lowering minocycline’s breakpoint to ≤1 mg/L reduced susceptibility from 77 to 35%, questioning its reliability (Veeraraghavan and Walia, 2025). Combination therapy (e.g., TMP-SMX with levofloxacin) may enhance efficacy (Banar et al., 2023). For CLABSIs (65.0% of isolates), catheter removal is critical, highlighting the challenges of addressing catheter-associated infections (Pfaller et al., 2023). Rising TMP-SMX resistance and CLSI’s 2025 recommendation against monotherapy necessitate MIC-based, genomics-informed approaches (Veeraraghavan and Walia, 2025). Enhanced infection prevention, including catheter care and stewardship, and research into resistance determinants are critical for managing this pathogen (Gupta et al., 2018; Li et al., 2023). Our results align with global surveillance showing sustained susceptibility of *S. maltophilia* to levofloxacin and minocycline, despite variable resistance to TMP-SMX. Recent studies reported over 90–95% susceptibility to these agents (Pfaller et al., 2023; Bostanghadiri et al., 2024), consistent with our findings. The revised CLSI breakpoint for minocycline (≤1 mg/L) has, however, led to a drop in reported susceptibility in several studies (Veeraraghavan and Walia, 2025), emphasizing the need for harmonized interpretive criteria. The observed shift from CLABSI to non-CLABSI infections may reflect improved catheter care and greater recognition of alternative infection sources, as also noted in multicountry ICU reports (Tanuma et al., 2025; Hase et al., 2024). The COVID-19 period likely influenced *S. maltophilia* infection patterns through increased antibiotic use and ICU strain. Global data indicate higher empirical antibiotic consumption and rising resistant Gram-negative infections during the pandemic (Sapula et al., 2024; Mosiun et al., 2025). This may have contributed to the increase in non-CLABSI events in later years of surveillance. These findings underscore the importance of strengthened antimicrobial stewardship and continuous national surveillance integrating infection-control data.

## 5 Limitations

Our dataset does not allow direct causal inference regarding the COVID-19 period; and future surveillance should specifically evaluate pandemic-related effects.

## Data Availability

The raw data supporting the conclusions of this article will be made available by the authors, without undue reservation.
