# Supplementary material for: Multicenter epidemiology of Stenotrophomonas maltophilia bloodstream infections in Indian ICUs: building digital surveillance network
Source: Front Microbiol. 2025 Dec 4;16:1725629. doi: 10.3389/fmicb.2025.1725629 (PMC12711849; doi:10.3389/fmicb.2025.1725629)
Supplement: Supplementary file 1 [file Table_1.pdf]

Supplementary Tables

Supplementary Table S1: Region-wise hospitals and medical institutions within the HAI Surveillance Network that reported cases of *Stenotrophomonas maltophilia* infection over a period of 7 years (2017-2024).

Region(s)	Hospital Name	No. of cases (n = 235)	No. of organisms (n = 271)
Central	AIIMS, Bhopal, Madhya Pradesh	8	10
	AIIMS, Raipur, Chhattisgarh	12	12
Northern	AIIMS, Bathinda, Punjab	4	4
	AIIMS, Jodhpur, Rajasthan	2	3
	AIIMS, New Delhi	45	47
	AIIMS, Rishikesh, Uttarakhand	4	4
	Dr. Baba Saheb Ambedkar Hospital, New Delhi	3	3
	KGMU, Lucknow, Uttar Pradesh	5	8
	MGMCH, Jaipur, Rajasthan	13	18
	PGIMER, Chandigarh	29	38
	SKIMS, Srinagar, Jammu and Kashmir	3	3
	Safdarjung Hospital, New Delhi	8	8
	SGRH, New Delhi	13	17
	HBCH & MPMMCC, Varanasi, Uttar Pradesh	1	1
Southern	AIMS, Kochi, Kerala	8	12
	Apollo Hospitals, Chennai, Tamil Nadu	3	3
	CMC, Vellore, Tamil Nadu	14	15
	KMC, Manipal, Karnataka	9	10
	NIMS, Hyderabad	3	3
Eastern	AIIMS, Bhubaneswar, Odisha	4	4
	IPGMER, Kolkata, West Bengal	15	17
	Institute Of Medical Sciences & Sum Hospital, Bhubaneswar, Odisha	1	1
	TMC, Kolkata	11	13
Western	AFMC, Pune, Maharashtra	1	1
	B. J. Medical College, Ahmedabad	4	4
	Government Medical College, Aurangabad, Maharashtra	1	1
	P. D. Hinduja Hospital, Mumbai, Maharashtra	8	8
North-Eastern	Agartala Government Medical College, Agartala, Tripura	1	1
	Assam Medical College and Hospital, Dibrugarh, Assam	1	1
	Regional Institute of Medical Sciences, Imphal, Manipur	1	1

Supplementary Table S2: Classification of ICUs reporting *Stenotrophomonas maltophilia* infections in the HAI Surveillance network from 2017 to 2024.

ICUs	Events {n (%)}	Organisms {n (%)}
Medical	51 (21.79)	62 (22.88)
Medical/Surgical	50 (21.37)	59 (21.77)
Trauma	28 (11.97)	31 (11.44)
Surgical	27 (11.54)	29 (10.7)
Pediatric	18 (7.69)	18 (6.64)
Neonatal	11 (4.68)	12 (4.43)
COVID	10 (4.27)	11 (4.06)
Gastrointestinal	6 (2.56)	10 (3.69)
Oncologic Medical	8 (3.42)	9 (3.32)
Cardiothoracic	6 (2.56)	6 (2.21)
High Dependency Unit	6 (2.56)	6 (2.21)
Oncologic Surgical	3 (1.28)	5 (1.85)
Anaesthesia	4 (1.71)	4 (1.48)
Neuro Surgery	3 (1.28)	4 (1.48)
Neurologic	1 (0.43)	2 (0.74)
Burn	1 (0.43)	1 (0.37)
Pediatric Medical/Surgical	1 (0.43)	1 (0.37)
Respiratory	1 (0.43)	1 (0.37)
Total	235	271

Supplementary Table S3: Region-wise classification of BSI events caused by *Stenotrophomonas maltophilia* over a period of 07 years (2017-2024).

Regions	Classification	No. of cases/events (n = 235)
Northern (n = 130)	CLABSI	79 (33.6)
	Non-CLABSI	40 (17.0)
	Secondary	11 (4.7)
Southern (n = 37)	CLABSI	28 (11.9)
	Non-CLABSI	7 (3.0)
	Secondary	2 (0.9)
Eastern (n = 32)	CLABSI	19 (8.1)
	Non-CLABSI	8 (3.4)
	Secondary	4 (1.7)
Western (n = 13)	CLABSI	6 (2.6)
	Non-CLABSI	6 (2.6)
	Secondary	2 (0.9)
North-Eastern (n = 3)	CLABSI	1 (0.4)
	Non-CLABSI	2 (0.9)
Central (n = 20)	CLABSI	16 (6.8)
	Non-CLABSI	3 (1.3)
	Secondary	1 (0.4)

Supplementary Table S4: Heat-Map depicting year-wise distribution of BSIs caused due to *Stenotrophomonas maltophilia* in various regions of India over a period of 7 years (2017-2024).

Year wise category	Central Region	Eastern Region	North-Eastern Region	Northern Region	Southern Region	Western Region	Total Isolates (n = 271)
2017-18	0	0	0	19 (70.4)	7 (25.9)	1 (3.7)	27
2018-19	1 (3.2)	2 (6.4)	1 (3.2)	20 (64.5)	7 (22.6)	0	31
2019-20	5 (14.3)	5 (14.3)	0	18 (51.4)	7 (20.0)	0	35
2020-21	3 (7.9)	5 (13.2)	1 (2.6)	14 (36.8)	11 (28.9)	4 (10.5)	38
2021-22	3 (8.1)	7 (18.9)	0	19 (51.3)	7 (18.9)	1 (2.7)	37
2022-23	0	8 (29.6)	1 (3.7)	15 (55.6)	2 (7.4)	1 (3.7)	27
2023-24	10 (13.2)	8 (10.5)	0	49 (64.5)	2 (2.6)	7 (9.2)	76

The colors in the table show the cases reported across regions in different years. The color intensity represents the percentage of cases, with green representing 0 cases, which intensifies through yellow, orange and reaches red, which represents the maximum recorded percentage of cases (i.e. 70.4%). The colors ranging from green to red represent increasing burden of cases.

Supplementary Table S5: Region-wise resistance pattern of *Stenotrophomonas maltophilia* against various drugs.

	Central Region	Eastern Region	North-Eastern Region	Northern Region	Southern Region	Western Region	TOTAL
TMZ (%R)	6/18 (33.3)	8/21 (38.1)	0/1	9/93 (9.7)	5/38 (13.2)	2/12 (16.7)	30/183 (16.4)
MINO (%R)	1/15 (6.7)	1/26 (3.8)	0/1	3/100 (3.0)	0/9	2/12 (16.7)	7/163 (4.3)
LEVO (%R)	3/21 (14.3)	5/29 (17.2)	0/1	7/141 (5.0)	1/41 (2.4)	2/11 (18.2)	18/244 (7.4)
TIG (%R)	0	0/1	0/2	1/7 (14.3)	1/7 (14.3)	0	2/17 (11.8)
COL (%R)	0	1/4 (25.0)	0	2/4 (50.0)	1/5 (20.0)	0/1	4/14 (28.6)
